# Space Jam‐Ming: Generating Interstitial Space Using Fragmented Granular GelMA to Investigate Novel Paradigms of Cancer Metastasis

**DOI:** 10.1002/advs.77478

**Published:** 2026-08-30

**Authors:** Danielle Vahala, Zhuang Min Lee, Sebastian E. Amos, Dong Woo Son, Jong Hwa Byun, Farzan Navaeipour, Jiayue Li, Kai L. Metzner, Ji Hoon Jeong, Juyeon Kim, Yongsung Hwang, Brendan F. Kennedy, Khoon S. Lim, Hee Seung Lee, Hyun Woo Park, Yu Suk Choi

**Affiliations:** ^1^ School of Human Sciences The University of Western Australia Perth Australia; ^2^ Medical School The University of Western Australia Perth Australia; ^3^ Department of Biochemistry Yonsei University Seoul Republic of Korea; ^4^ BRITElab Harry Perkins Institute of Medical Research Perth Australia; ^5^ Department of Electrical Electronic and Computer Engineering School of Engineering The University of Western Australia Perth Australia; ^6^ Soonchunhyang Institute of Medi‐Bio Science Soonchunhyang University Chungcheongnam‐do Republic of Korea; ^7^ Institute of Physics Faculty of Physics Astronomy and Informatics Nicolaus Copernicus University in Toruń Toruń Poland; ^8^ School of Medical Sciences University of Sydney Sydney Australia; ^9^ Division of Gastroenterology Department of Internal Medicine College of Medicine Yonsei University Seoul Republic of Korea

**Keywords:** cancer invasion, cell‐ECM adhesion, confinement, microgels, organoid

## Abstract

The tumor microenvironment undergoes extensive remodeling during cancer progression, resulting in increased collagen, altered tissue mechanics, and the formation of collagen tracks that permit migration. However, how the extracellular matrix (ECM) regulates cellular plasticity remains less known. Cellular plasticity is essential for successful metastasis, as cells undergo epithelial‐to‐mesenchymal transition and adherent‐to‐suspension transition (AST). Studies have begun to use 3D photo‐crosslinkable hydrogels, but, unlike in vivo ECM, hydrogel stiffness is inextricably linked to porosity. In this study, we propose a fragmented gelatin methacryloyl (GelMA) scaffold that controls stiffness independently from porosity. When encapsulated as single cells, non‐metastatic breast cancer cells do not exhibit growth restriction, whilst pre‐engineered metastatic breast cancer cells show altered mechanosensitivity and enhanced migration (*p* < 0.05). We next study the role of AST in cellular migration and observe similar velocity to invasive metastatic cells. Interestingly, non‐metastatic cells showed AST‐dependent migration within interstitial spaces, which was enhanced in the stiff scaffold (*p* < 0.05). AST induction significantly increased Lamin A/C (associated with providing nuclear stability for circulating tumor cells) and reduced nuclear yes‐associated protein (YAP) (necessary for cell detachment). Our data highlights the importance of incorporating micro‐scale porosity and presents a promising platform for the study of cellular growth and migration.

## Graphical abstract

Microgels enable independent control of stiffness and interstitial space, overcoming limitations of conventional 3D hydrogels. Using fragmented gelatin methacryloyl microgels, we show that stiffness does not restrict non‐metastatic breast cancer growth, contrary to bulk hydrogels, and reveal the role of adherent‐to‐suspension transition in cancer metastasis.

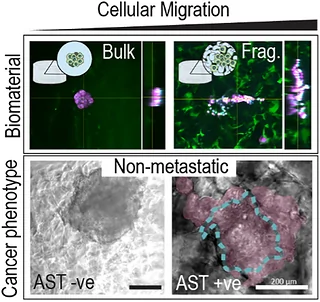

## 1 Introduction

Metastasis, the main cause of cancer‐related death, remains an inexorable process wherein cancer cells adopt the cellular plasticity required to overcome the ubiquitous mechanical challenges posed by the tumor microenvironment (TME) [1, 2, 3]. Advancements in biomaterial development, including photo‐crosslinkable hydrogels, have enabled 3D culture and stiffness tuneability, allowing investigation into TME‐regulated cancer cell fate. Most photo‐crosslinkable hydrogels, although highly tuneable, present mechanical coupling between stiffness and porosity that differs from the in vivo environment [4]. As a result, stiffer hydrogels may impose mechanical confinement through reduced pore size, potentially confounding the interpretation of 3D cancer cell behavior. This has been demonstrated extensively through viscoelastic hydrogels which offer stress relaxation and restore cell spreading, proliferation, migration, differentiation, and organoid formation [5, 6]. In this study, we aim to develop a new hydrogel platform that offers greater biomimicry of the extracellular matrix (ECM), specifically the micron‐sized pores within the interstitial spaces [7, 8], whilst maintaining microgel properties and stiffness tuneability. By engineering microenvironments with independently tuneable stiffness and microscale pore network, we can dissect the distinct contributions of TME mechanical properties regulating cellular growth and migration.

**FIGURE 1:**
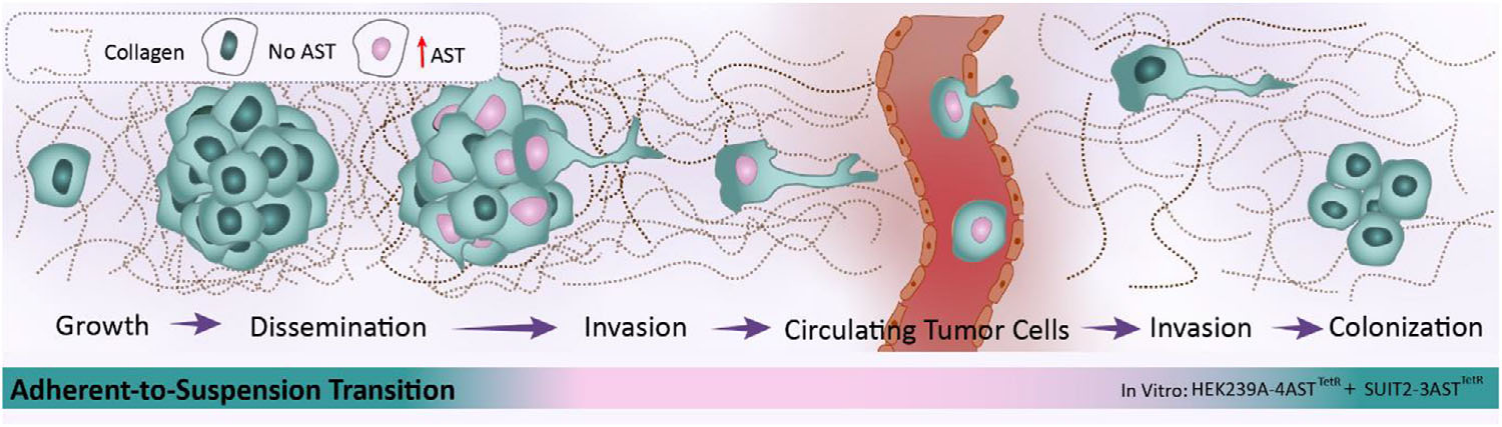
Adherent‐to‐suspension transition (AST) may contribute to multiple stages of the metastatic cascade and occur in complement to the epithelial‐to‐mesenchymal transition (EMT). Herein, we propose AST as an alternative paradigm that is necessary for the completion of the entire metastatic cascade. Its involvement in early metastasis is still largely unknown and will be explored in this study. Inducible cells using the TetR system (HEK293A‐4AST^TetR^ and SUIT2‐3AST^TetR^) were used to study the transitional role of AST in non‐metastatic and metastatic origins, respectively.

The most widely accepted paradigm describing cancer cell plasticity during tumor dissemination is the epithelial‐to‐mesenchymal transition (EMT) which enables epithelial cells to undergo a series of biochemical changes (downregulation of E‐cadherin, loss of cell‐cell contacts and polarity) ultimately resulting in a mesenchymal phenotype with enhanced migratory capacity [9]. However, recent reports have correlated increased metastatic capacity and poor prognosis with sustained E‐cadherin expression and collective cell migration [10, 11]. These observations have prompted the exploration of alternative frameworks that may capture key features of metastasis, including “cell‐cell/ECM adhesion” and “suspension under shear stress” [12]. Unlike EMT, adherent‐to‐suspension transition (AST) has proven vital for the transition to and survival of circulating tumor cells (CTCs) [13]. Through the induction of 4 or fewer hematopoietic transcription factors including IKAROS family zinc finger 1 (IKZF1), nuclear factor erythroid 2 (NFE2), intergeron regulatory factor (IRF8), and BTG anti‐proliferation factor 2 (BTG2), cells can be directly converted from adherent phenotype (A) to suspended phenotype (S), gaining anoikis resistance and reduced anchorage dependency [14]. Overall, AST has proven pivotal for the successful completion of cancer metastasis and has begun to fill in the gaps left by EMT alone (Figure 1). In the discovery of the AST paradigm, CTC formation was found to be dependent on successful AST [15]. More recently, expression of AST‐associated genes was identified in highly metastatic CTC populations located both within the perivascular regions of the primary tumors and distance metastatic sites [16]. Importantly, the AST transcriptional program has been observed across multiple cancer types, independent of specific oncogenic drivers, suggesting a broadly conserved mechanism of metastatic dissemination [16].

To date, AST has been primarily studied in relation to CTCs^13^ and its role in early stages of metastasis [15], including primary tumor growth, dissemination, and local invasion, is largely unknown, even though recent reports observe expression in peripheral regions of primary tumor [16]. To study these transient mechanisms in the TME, it is essential to employ biomaterials that enable their investigation. In a previous study [17], we showed that mechanical coupling of stiffness and the pore network in bulk hydrogels can lead to restricted volumetric expansion of breast cancer spheroids in a stiff environment. This highlights how limitations in biomaterial design can unintentionally perturb normal cellular processes. In the case of photo‐crosslinkable hydrogels, this limitation arises from a restricted ability to independently control porosity. To overcome this challenge, granular hydrogels have emerged as an exciting new class of biomaterials that offer the stiffness tuneability of photo‐crosslinkable hydrogels while providing control over interstitial spaces that more closely resemble in vivo tissue architecture. Numerous methodologies have been developed for generating microgels [18, 19, 20], which can then be “jammed” to create a stable scaffold for encapsulation of cells within a micro‐porous network. The properties of the microgels, and subsequent granular scaffolds, will be reliant on the method of fabrication [18, 19, 20]. We chose granular gelatin methacryloyl (GelMA) using the fragmentation method as it recapitulates the heterogeneity of the TME. Moreover, the heterogeneity of fragments can generate interstitial spaces resembling collagen tracks generated in the remodeled TME that serve as highways to lymph and blood vessels for cancer cells [21, 22]. Successful methods have previously been developed to control confinement with tunable stiffness via microfabrication techniques [23]. Herein, we propose a GelMA‐based technique as an alternative approach to mechanistically dissect stiffness from spacing, which may prove useful for researchers studying cell migration.

## 2 Results and Discussion

### 2.1 3D Granular Hydrogels for Mimicking Tumor Microenvironment

The delicate interplay between the microenvironment and cell fate is complex and dynamic, requiring tuneable biomaterials that can recapitulate key features of native tissues. Previous measurements using atomic force microscopy (AFM) measured healthy breast tissue as 1.13 ± 0.78 kPa, whereas breast tumors were broadly distributed up to ∼20 kPa [24]. To mimic this range of stiffnesses we utilized GelMA [25, 26, 27], a versatile polymer that enables control of cross‐linking (and thus stiffness) via alterations in polymer concentration or photoenergy. This tuneability, although suitable for mimicking tissue stiffness, is inextricably linked with the density of the pore network [26]. Consequently, cells encapsulated within a soft and stiff hydrogel will sense not only the stiffness, but also the difference in pore network.

**FIGURE 2:**
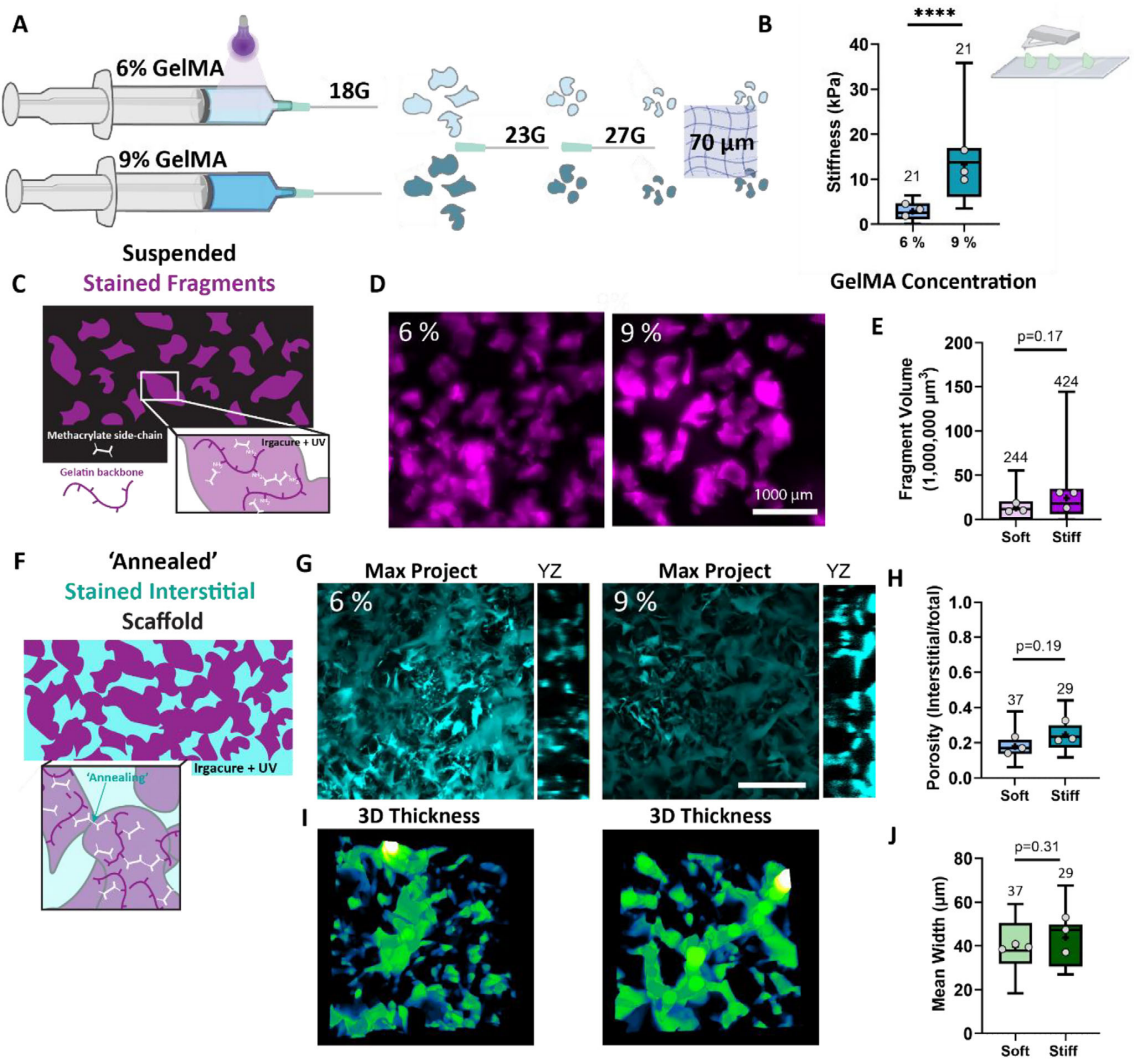
Fragmentation of gelatin methacryloyl (GelMA) enabled the formation of granular scaffolds of varying stiffness but equal macro‐porous interstitial spaces. (A) 6% and 9% GelMA were polymerized within a syringe and extruded through a series of gauges (18G, 23G, 27G) and a 70 µm pore cell strainer. (B) AFM was conducted on two concentrations of GelMA microgels, soft 2.9 ± 0.4 kPa (6%, *n* = 21) and stiff fragments 13.3 ± 1.8 kPa (9%, *n* = 21) (*p* < 0.0001). (C) Polymerization within the syringe generates intra‐particle polymerization where some of the methacrylate side‐chains cross‐link via free‐radical polymerization. (D) FITC‐Dextran was added to the microgel solution (artificially colored magenta) to enable immunofluorescence‐based morphological analysis of GelMA fragments. Scale bar represents 1000 µm. (E) Microgel volume was calculated for both 6% (soft; *n* = 244) and 9% (stiff; *n* = 424) GelMA fragments. (F) After jamming via centrifugation, inter‐particle polymerization is generated by cross‐linking of spare methacrylate side‐chains between neighbouring particles (in the presence of UV and Irgacure). (G) Adding FITC‐Dextran to the mounting glycerol enabled visualization of interstitial space (artificially colored cyan). Scale bar represents 200 µm. (H) We calculated porosity as a measurement of interstitial volume/total volume. (I) Thickness plots were generated via the BoneJ Fiji Thickness plugin (J), and we calculated average widths per scaffold (*p* > 0.05, *n* = 37 and 29 for soft and stiff scaffolds, respectively). Box plots represent individual points, and Student's *t*‐tests were performed using the mean of each independent biological repeat (grey dots, *n* = 3).

To address this limitation, GelMA hydrogels were fragmented post‐polymerization via an extrusion‐based method to generate microgels (Figure 2A) [18, 28]. To span physiologically relevant stiffnesses (previously reported to be broadly distributed up to 20 kPa) [24] 6% and 9% GelMA concentrations were selected, yielding fragment stiffnesses of 2.9 ± 0.4 kPa and 13.3 ± 1.8 kPa, respectively (Figure 2B). Importantly, cancer progression is accompanied by enhanced collagen deposition, which results in both increased collagen density (ligand presentation) and stiffness. For this reason, we chose to use different GelMA concentrations with uniform photo‐energy.

Fluorescein isothiocyanate‐dextran (FITC‐Dextran) labelling enabled 3D visualization (Figure 2C) and quantification of fragment morphology, confirming no difference in fragment volume between conditions (Figure 2D,E). Fragments could then be “jammed” (via centrifuge) and polymerized a second time to cross‐link free methacryloyl side‐chains, generating stable scaffolds with microscale pores (Figure 2F and Figure S1A). These scaffolds maintained mechanical stability over time (Figure S1B), and heterogenous composition could be generated by combining fragments of differing stiffness (Figure S1A,C). Elastography maps further confirmed distinct 3D stiffness profiles across scaffold conditions (Figure S1D).

We next investigated the most important feature of this platform, the microscale pores, which was quantified as the interstitial volume between fragments and visualized through FITC‐Dextran (Figure 2G). Although fragmentation produced a broad distribution of pore sizes (Figure S2), overall porosity remained consistent across scaffolds of differing stiffness (Figure 2H). Analysis of interstitial space using the BoneJ Thickness function enabled quantification and visualisation of pore width distributions within soft and stiff GelMA scaffolds (Figure 2I,J). There was no significant difference in average pore width between soft (39.72 ± 12.21 µm) and stiff (43.84 ± 3.04 µm) scaffolds (*p* = 0.31). To capture the heterogeneity of the scaffolds, we pooled histograms and observed a broad range of thicknesses from ∼1 to 160 µm (Figure S2A). The larger reported pores may be an artifact of the spheroids encapsulated within the scaffold which were pre‐engineered to be ∼150 µm in size. Importantly, cells will experience different degrees of confinement, and for this we compared the minimum width generated within the soft (1.94 ± 0.5 µm) and stiff (2.2 ± 0.8 µm) scaffolds (Figure S2B). Importantly, native collagen tracks generated by MDA‐MB‐231 cells within collagen matrices have been reported to exhibit widths of approximately 13.7 µm [7]. Therefore, to better understand the pore distribution we calculated the proportion of clefts that fall below 15 µm, which was 46% and 42% for soft and stiff, respectively (Figure S2C). Moreover, in vivo studies of collagen track formation similarly report initial track widths of approximately 15 µm, which expand to 60–80 µm following cellular infiltration [8]. In the granular GelMA scaffold 85% and 82% (soft and stiff) of clefts fall below 80 µm (Figure S2C), reinforcing the scaffold as a useful tool for studying cellular migration through a remodeled TME. Collectively, these findings, together with supporting literature, suggest that the fragmented granular GelMA platform recapitulates the heterogenous interstitial spacings observed after remodeling occurs in vivo. To examine whether porosity could be modulated by altering packing conditions we generated scaffolds through vacuum jamming or centrifuge jamming; which resulted in enhanced and reduced porosity, respectively (Figure S3A,B). Together, these results establish a granular scaffold with independently tuneable microgel properties and controllable porosity.

### 2.2 Granular Scaffolds Enable Cancer Modeling In Vitro for Both Primary Tumor Growth (MCF7) and Local Migration (MDA‐MB‐231)

**FIGURE 3:**
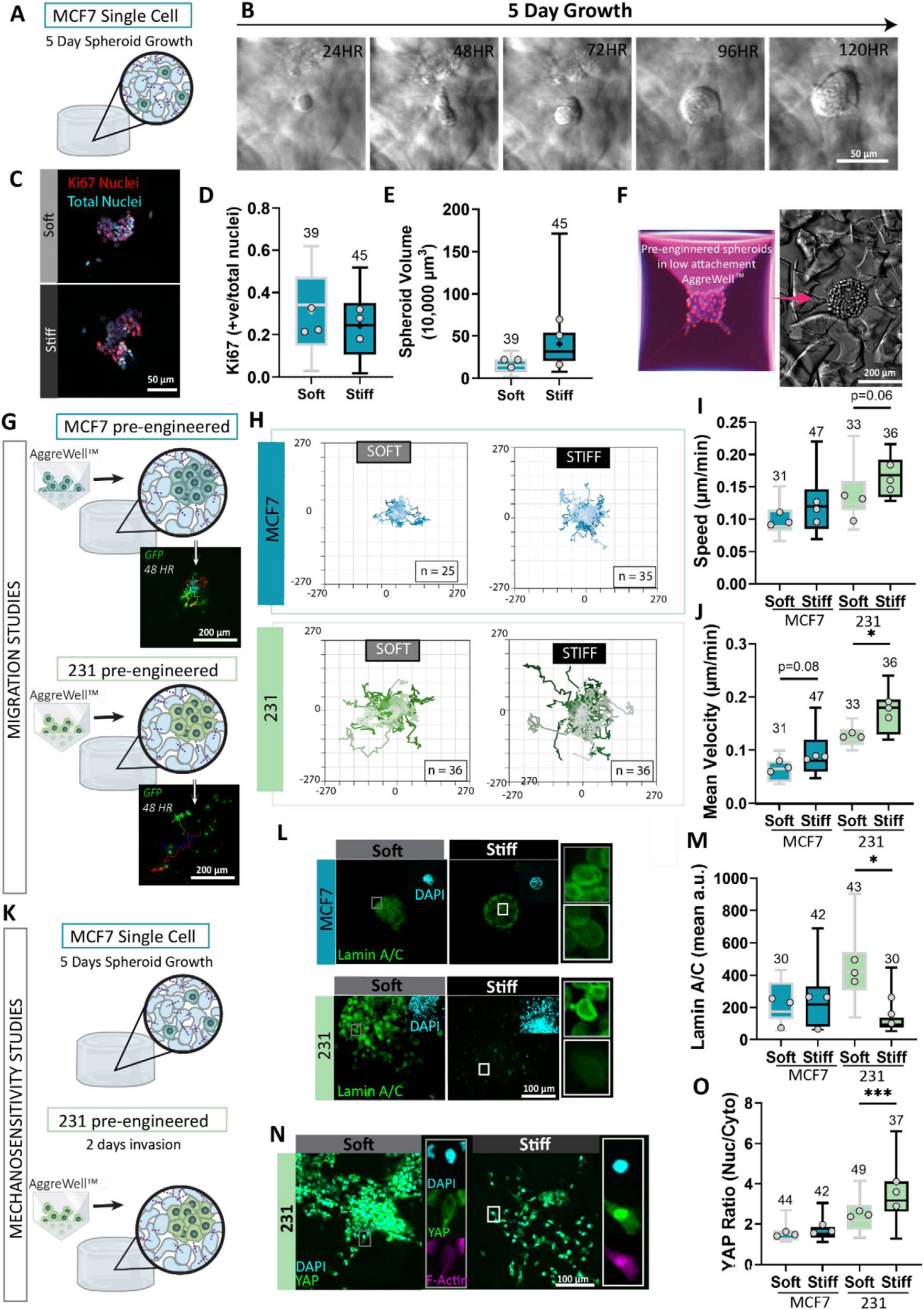
Granular GelMA scaffold with tuneable stiffness (soft 2.9 kPa and stiff 13.3 kPa) enabled phenotype‐dependent growth or migration as modelled by non‐metastatic MCF7 (cyan) and highly metastatic MDA‐MB‐231 (231, green). (A) To model tumor growth, MCF7 cells were embedded as single cells within the granular scaffold and (B) left to culture over 5 days. (C and D) We stained for the Ki‐67 proliferative marker, and (E) measured final spheroid volume (total number of spheroids; *n* = 39 and 45 in soft and stiff, respectively). (F) To model migration, 231 and MCF7 cells were pre‐engineered as 100 cells per spheroid using an AggreWell, prior to encapsulation within granular scaffolds. Spheroids were added to the microgel suspension and “jammed” within the scaffold via centrifugation. The scaffold underwent a second UV polymerization to produce a stable scaffold with embedded spheroids within interstitial spaces. (G) For 48 h, H2B‐GFP nuclei were captured at 15‐min intervals to track (H) migration via TrackMate [31] (lines represent nuclear trajectories). Migration was quantified through speed (I) and velocity (J) (*p* < 0.05). (K) For investigating mechanosensitive proteins (Lamin A/C and YAP), the growth model was used for MCF7 (single‐cell embedding and 5 days culture) and the migration model for 231 (pre‐engineered spheroid and 2 days culture). (L–O) Immunocytochemistry was performed to quantify mechanosensitive proteins Lamin A/C (*p* < 0.05) and YAP expression. Box plots represent individual points (number reported above bars) and unless stated a Kruskal–Wallis test or unpaired nonparametric *t*‐test were performed (on non‐parametric data) using the mean of each independent biological repeat (grey dots, *n* = 3).

To validate GelMA granular scaffolds as a tool to study primary tumor growth and local migration, we tested two widely used breast cancer cell lines, MCF7 and MDA‐MB‐231 (231), which possess non‐metastatic and metastatic phenotypes, respectively. These models are frequently employed to investigate the epithelial‐to‐mesenchymal transition. A previous study has investigated the role of stiffness during early tumor growth using MCF7 spheroids within a linear stiffness gradient hydrogel platform; however, this system lacked micro‐scale porosity [17] and imposed mechanical confinement on spheroids [29]. Consequently, leading to a mechanically confined phenotype, which was further confirmed. Notably, even when bulk hydrogels were tuned to match breast tumor stiffness, local migration of highly metastatic 231 cells remained limited (Figure S4A). In this study, we embedded individual MCF7 cells within the interstitial spaces of the granular GelMA scaffold (Figure 3A). Encapsulated MCF7 cells depicted steady growth into spheroids over 5 days (Figure 3B). Proliferation and spheroid size were unchanged across scaffolds with matched porosity but differing stiffness (*p* > 0.05) (Figure 3C–E). This contrasts with mechanically coupled hydrogel systems, where stiffness has been associated with reduced spheroid expansion [17]. Together, these results establish a granular scaffold with tuneable porosity independent of microgel properties, i.e., stiffness and ligand concentration.

In cancer, migration of cells has been observed to occur within remodeled collagen tracks, which offer greater porosity than the surrounding dense matrix while being stiffer [21]. This system provides a pre‐formed microporous network that mimics collagen tracks observed in vivo [7]. To model migration, pre‐engineered spheroids were embedded within a granular scaffold (Figure 3F). Over 48 h, 231 cells exhibited extensive dissemination from spheroids, whereas pre‐engineered MCF7 spheroids remained largely intact (Figure 3G,H). In contrast, 231 spheroids cultured in traditional bulk hydrogels showed minimal migration despite comparable microgel conditions (Figure S4A). Both cell types exhibited a trend toward increased migration in stiffer scaffolds, however only 231 cells had significantly enhanced velocity (1.38‐fold increase, *p* < 0.05) (Figure 3I,J) [30]. MCF7 cells, despite showing slight increase in motility in stiffer scaffold, did not disseminate away from the spheroid even after 9 days of culture (Figure S4B). Together, these results suggest that fragmented granular hydrogels are useful tools for investigating cell migration, as they provide interstitial space for dissemination while relying on the phenotypic capacity of cells to migrate through the scaffold. This is especially important for studying cancer plasticity, where enabling and distinguishing between in vivo phenotypes is essential. It should be noted the relatively large variability observed between spheroids likely reflects, at least in part, the heterogenous interstitial architecture of the fragmented GelMA scaffold, where pore widths span approximately 1–160 µm. While this heterogeneity increases variability in individual cellular responses, it also represents an inherent characteristic of the fragmented model, which was designed to capture irregular interstitial spaces rather than uniform pore network. These findings probe further investigation into MCF7 cells within 3D microenvironments, which differ substantially from the uniform mechanical conditions typically investigated in 2D culture.

Next, we wanted to explore whether mechanosensitivity was altered when we controlled stiffness independent of porosity. Lamin A/C is responsible for maintaining nuclear stability and Yes‐associated protein (YAP) is a transcription factor that translocates under stiffening conditions. Previously, MCF7 was reported to have differential expression of YAP and Lamin A/C across the stiffness gradient, however, this was in a mechanically confined bulk hydrogel [17]. Here, we use the granular platform with the previous MCF7 growth model (single‐cell to spheroid growth over 5 days) and 231 migration model (pre‐engineered spheroid migration over 4 days) (Figure 3K). MCF7, which presented no differences in growth (Ki‐67 and volume in Figure 3D,E), consistently showed no differences in Lamin A/C or nuc/cyto YAP when cultured in soft or stiff granular scaffolds (Figure 3L–O).

In contrast, 231 cells cultured in stiff scaffolds exhibited increased nuclear translocation of YAP (Figure 3N,O), consistent with a mesenchymal mechanotransduction response (*p* < 0.001), which was unexpectedly accompanied by reduced Lamin A/C expression (2.94‐fold decrease, *p* < 0.05) (Figure 3L,M), challenging the traditional mechanotransduction paradigm. Recent investigations in confined 3D environments have similarly reported downregulation of Lamin A/C expression under mechanically restrictive conditions [32, 33], with low Lamin A/C expression associated with enhanced metastatic potential and increased nuclear deformability. Consistent with these findings, in our study, 231 cells cultured within a stiff, 3D GelMA matrix exhibited reduced Lamin A/C alongside increased migratory behavior. There are reports of phenotypic heterogeneity within primary tumors, with more aggressive “metastatic” cells typically found within the leading edge, or periphery of the tumor [34]. For this reason, we sought to quantify the spatial expression of Lamin A/C (i.e., core‐periphery) (Figure S5A). Peripheral cells showed consistently lower Lamin A/C expression compared to the core (Figure S5B). Moreover, it is known that YAP, through the Hippo pathway, drives cytoskeletal reorganisation to promote migration [35, 36, 37]. These findings suggest that reduced Lamin A/C and increased YAP activity together support a mechanically adaptive, migratory phenotype in stiffer (and more concentrated) environments. This recapitulates the well‐known notion that tumor progression (and spread of cancer) is accompanied by increased ECM mechanics (via enhanced collagen density and stiffness). Taken together, we propose that the interstitial space generated in this granular GelMA scaffold is of greater biological relevance to the in vivo tumor microenvironment and has successfully enabled the expression of binary cellular phenotypes (non‐metastatic and metastatic) of MCF7 and 231.

EMT is a dynamic process whereby cells undergo a series of phenotypic changes: loss of cell‐cell connections (loss of E‐cadherin), increased vimentin production, and reduced cell polarity. MCF7 and 231 cells represent the binary endpoints of this spectrum but lack the plasticity to dynamically undergo EMT or MET without further induction. Having validated the granular scaffold as a useful tool for studying different phenotypes, we next wanted to explore the dynamic process of EMT via transforming growth factor beta (TGFβ) treatment of MCF7 cultured spheroids [38, 39, 40]. Unfortunately, TGFβ treatment produced minimal changes in migration or mechanosensitive markers after 4 days of treatment (*p* > 0.05) (Figure S6A–D), indicating that EMT‐like transitions were not robustly recapitulated under these conditions.

### 2.3 AST Induction Within Non‐Metastatic Cells Enables Primary Spheroidal Growth and Promotes Migration

**FIGURE 4:**
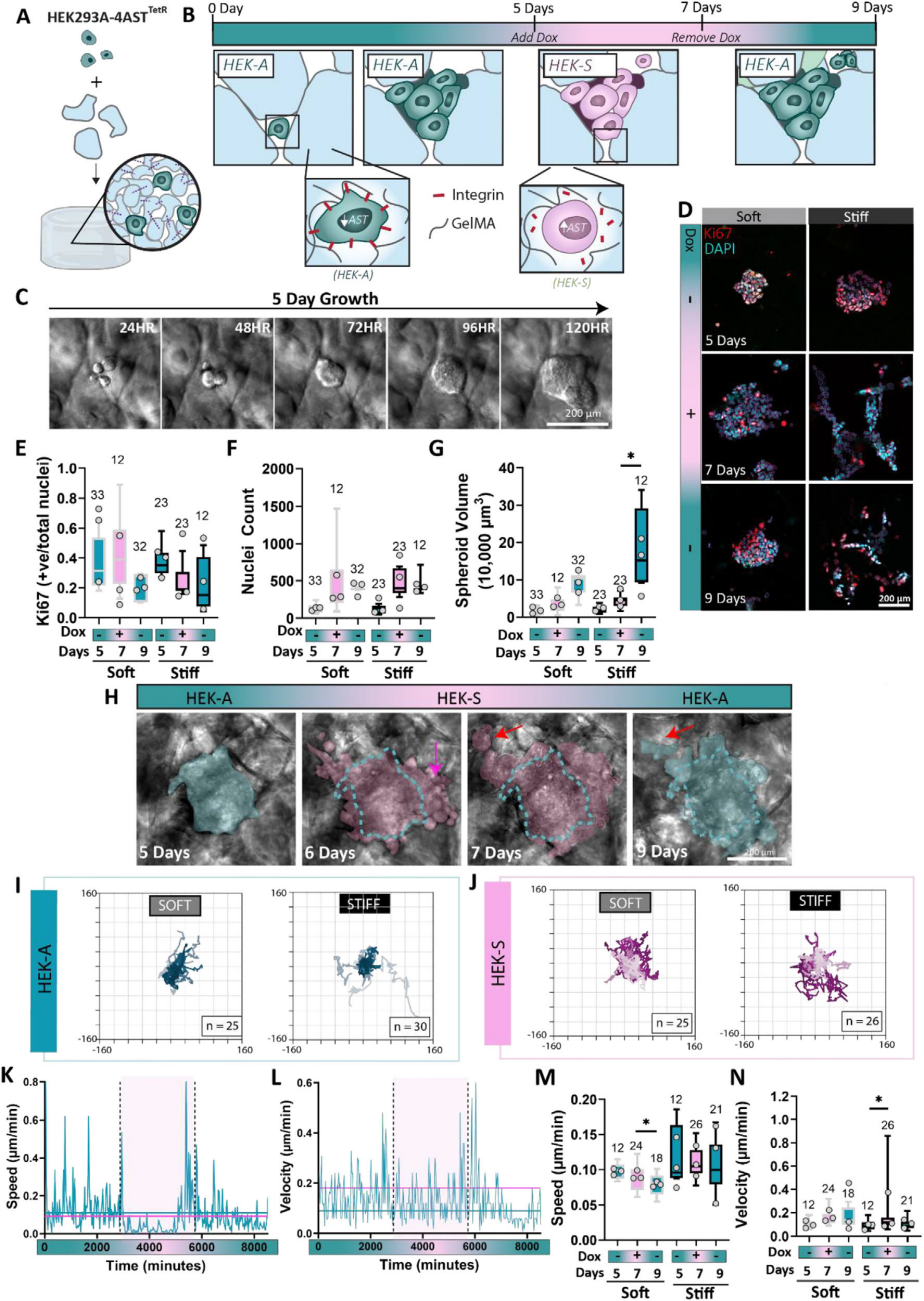
The fragmented granular scaffold did not show mechanically confined growth of AST‐inducible TetR non‐metastatic HEK293A‐4AST^TetR^ and enabled AST‐dependent migration away from the primary spheroid. (A) HEK293A‐4AST^TetR^ was seeded as single cells into either soft 2.9 ± 0.4 kPa (light grey) or stiff 13.3 ± 1.8 kPa (black) granular scaffolds. (B) The non‐metastatic adherent phenotype (HEK‐A, blue) maintained cell‐ECM connections via integrins (red dash) and generated spheroids after 5 days, at which point doxycycline (Dox) was added to induce the suspension phenotype for 48 h (HEK‐S, pink). Finally, Dox was removed for a further 48 h to reinduce the adherent phenotype (HEK‐A). (C) Successful growth of spheroids was monitored using live‐cell imaging over the 5‐day time course. (D and E) Growth was quantified using the proportion of Ki‐67‐positive cells and (F) nuclei count. (G) Spheroid volume was significantly larger in the stiffer scaffold (*p* < 0.05). H) In the presence of Dox, cells begin to individualize (pink arrow) and disseminate into the scaffold (red arrow). Dox is removed to investigate the fate of these disseminated cells after 48 h. (I and J) Tracking of H2B‐GFP‐tagged nuclei was conducted in soft (light grey) and stiff (black) fragmented GelMA and analysed through the Fiji plugin TrackMate [34] (*n* = 106). We tracked an individual cell to calculate perturbations in speed (K) and velocity (L) during the transitions from A to S and back to A (*n* = 1) and repeated this for the population of cells (M and N) (*p* < 0.05, *n* = 113). Box plots represent individual points (number reported above bars), and unless stated a Kruskal–Wallis test or unpaired nonparametric *t*‐test were performed (on non‐parametric data) using the mean of each independent biological repeat (grey dots, *n* = 3). Scale bar represents 200 µm.

The adherent‐to‐suspension transition (AST) is a novel paradigm, however, its molecular mechanisms were previously investigated in a study using HEK293A cells with 4 AST factors (IKZF1, BTG2, NFE2, and IRF8) in a doxycycline (Dox) inducible TetR system (HEK293A‐4AST^TetR^). This study identified loss of several integrins as a key defining feature [14]. In our system, GelMA presents Arg‐Gly‐Asp (RGD) motifs that enable integrin‐mediated adhesion through α5β1 and αVβ3 integrins. Interestingly, these key integrins are downregulated following AST induction (Figure S7A), suggesting a shift toward anchorage‐independent behavior. When cultured on a fibronectin‐coated (RGD‐presenting) 2D surface, AST induction resulted in cell detachment, further validating the loss of integrin‐mediated cell adhesion (Figure S7B). To study the role of AST on non‐metastatic cells during cell growth and migration, we used the same system previously explored [15]. This system enabled controlled switching between an adherent (HEK‐A) and suspended (HEK‐S) phenotype. Individual HEK293A‐4AST^TetR^ cells were encapsulated within either soft (∼2.9 kPa) or stiff (∼13.3 kPa) granular GelMA scaffolds (Figure 4A). Within the interstitial space of the granular scaffold, the transition from adherent (A) to suspension (S), and reverse S to A, was captured over the course of 9 days (Figure 4B). HEK‐A, which maintained cell‐cell and integrin‐ECM connections, showed successful spheroid growth over the first 5 days of culture (Figure 4C), similar to non‐metastatic cancer cells, MCF7. Spheroid growth was unaffected by scaffold stiffness, with no differences in proliferation (Figure 4D,E) or cell number (Figure 4F). This finding reaffirms observations from a previous study on non‐metastatic MCF7 cell growth, where introducing interstitial space eliminated the stiffness (and/or pore network) restricted growth typically seen in 3D bulk hydrogels [17]. Taken together, these results not only support the use of the granular platform as a valuable tool for tumor growth studies, but also suggest that AST induction in epithelial cells does not negatively impact tumor growth. We next examined nuclear morphology following AST induction and observed a reduction in nuclear volume from 650 to 547 µm^3^ (Figure S8A) and elongation (1.17‐fold) (Figure S8B), indicating transformation to smaller, rounder cells, which is suggestive of a loss of ECM interaction. Although these trends were not statistically significant, they align with the expected loss of adhesion (and shedding of integrins) associated with AST [14]. Interestingly, the largest spheroids were recorded in the stiff granular scaffold (*p* < 0.05) (Figure 4G). This trend was not accompanied by changes in proliferation or cell number, suggesting that it arises from altered cell organization rather than enhanced growth (Figure S8C). Taken together, we propose the increase in volume is not due to growth but rather more spreading, which is evident in low‐magnification imaging (Figure S8C). Overall, these results indicate that AST enables phenotypic transitions without compromising growth, while scaffold stiffness influences cell organization within the interstitial space.

After 5 days of spheroid growth, AST was induced via doxycycline treatment (Figure 4B), and cell behavior was monitored using live cell imaging. We observed the first signs of cells individualization within the spheroid (Figure 4H) after 24 h of Dox treatment (pink arrow) (Figure 4H), followed by complete individualization of cells from the spheroid after 48 h (red arrow) (Figure 4H). Even when cells did not completely dissociate from the primary spheroid, it was consistent that the surface of the primary spheroid “bubbles” as each cell's contours became more visible (green arrow) (Figure S9A and Video S1). Following removal of doxycycline, cells exhibited two distinct behaviors. Cells that remained in close proximity to the primary spheroid were reabsorbed (pink arrow) (Figure S9B and Video S2), whereas cells that migrated further away formed secondary spheroids (red arrow) (Figure S9C and Video S3A–C). In the absence of AST induction, no dissemination was observed, confirming this behavior was AST‐dependent (Figure S9D). The reabsorption of cells in close proximity to the primary spheroid is an interesting phenomenon. Examination of RNA sequencing data revealed that E‐cadherin expression decreased slightly following AST induction from 0.02 to 0.01 and N‐cadherin expression decreased from 17.2 to 14.6 (Figure S10). Therefore, SAT induction may partially restore cell–cell junctions through modest recovery of cadherins and cortical protein (β‐catenin / α‐catenin) expression (Figure S10). When doxycycline treatment was continued, AST+ve cells sustained individualized phenotype (as observed by appearance of cell contours) (Video S4). These observations indicate that AST enables reversible transitions between cohesive and dispersed states, supporting both dissemination and re‐aggregation.

Tracking H2B‐GFP‐tagged nuclei of HEK‐A and HEK‐S revealed increased migration in stiffer scaffolds and enhanced motility following AST induction (Figure 4I,J). At the single‐cell level, AST induction resulted in altered migration dynamics, characterized by reduced speed (pink line, Figure 4K) but increased directional persistence (velocity; pink line, Figure 4L). At the population level, cells exhibited modest increases (1.2‐fold) in motility in stiff scaffolds (*p* > 0.05) (Figure 4M), with AST‐expressing cells showing significantly enhanced velocity in the stiff scaffold (*p* < 0.05) (Figure 4N). This aligns with earlier results, which suggested more “spreading” (correlating to increased spheroid volume) and thus migration occurred in the stiff granular scaffold (Figure S6C). Given that non‐metastatic cells typically exhibit limited migratory capacity, AST may represent an alternative mechanism contributing to early dissemination in tumor progression.

### 2.4 AST Induction Did Not Impair the Migration of Metastatic Cancer Cells

**FIGURE 5:**
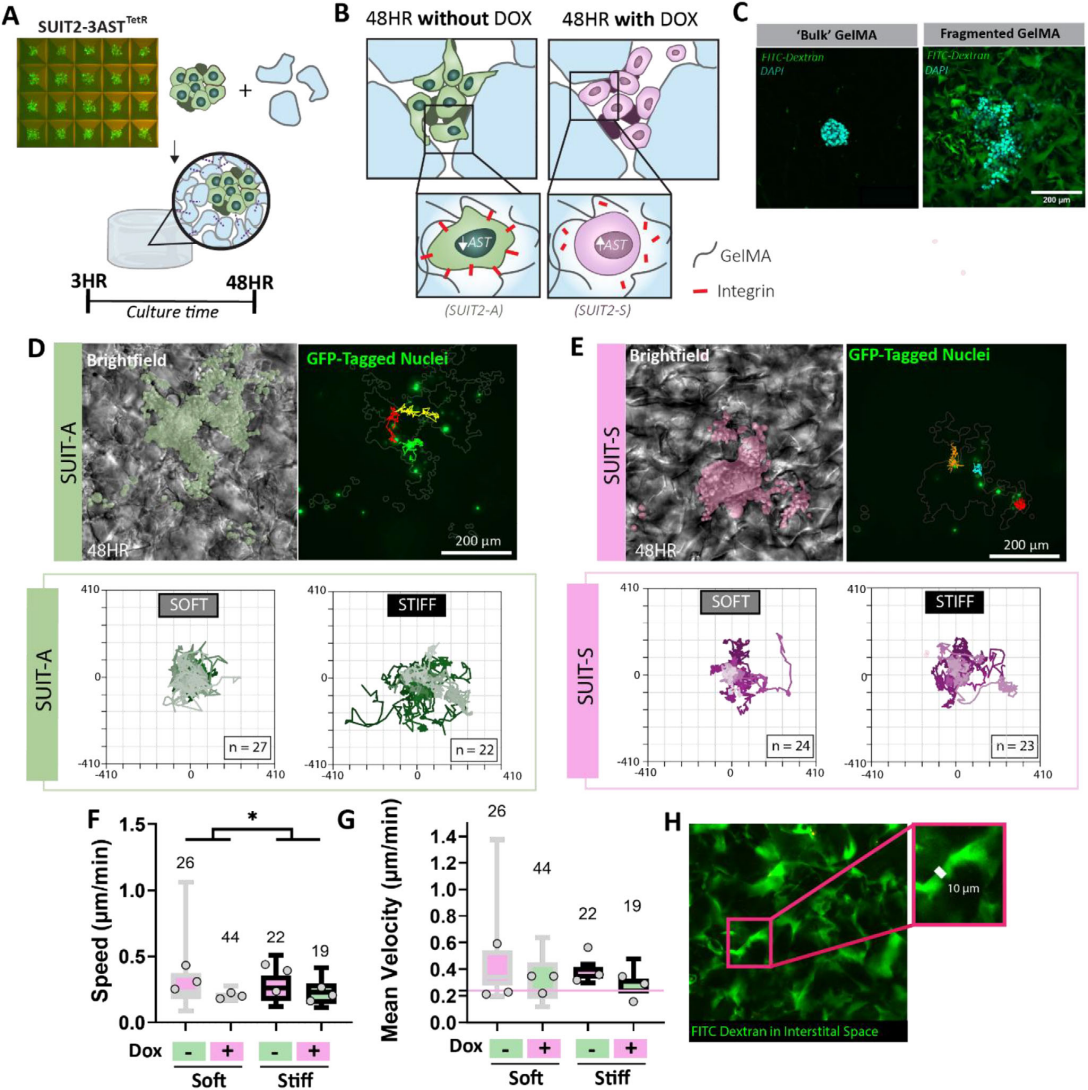
3D migration of metastatic SUIT2 in its adherent (SUIT2‐A, green) and suspended phenotypes (SUIT‐S, pink). (A) Pre‐engineered SUIT2‐3AST^TetR^ was added to soft (2.9 ± 0.4 kPa) and stiff (13.3 ± 1.8 kPa) GelMA scaffolds. (B) Within the interstitial spaces of the granular scaffold, we investigated migration for 48 h as adherent (*n* = 49, SUIT2‐A) phenotype (without Dox, green) or suspended (*n* = 47, SUIT2‐S) phenotype (with Dox, pink). (C) We compared SUIT2‐S morphology after embedding in traditional “bulk” GelMA hydrogel vs. fragmented GelMA. (D and E) Live‐tracking was conducted on H2B‐GFP‐tagged SUIT2‐A and SUIT2‐S cells. The speed (F, two‐way ANOVA, *p* < 0.05) and velocity (G) was calculated through TrackMate plugin. Stiffnesses are represented as either light grey border (soft) or black (stiff). (H) To measure the interstitial channels, FITC‐dextran was added to the mounting glycerol and left to permeate the scaffold. Scale bar represents 200 µm. Box plots represent individual points (number reported above bars) and unless stated a Kruskal–Wallis test or unpaired nonparametric *t*‐test were performed (on non‐parametric data) using the mean of each independent biological repeat (grey dots, *n* = 3).

To assess the role of AST in metastatic cells, we utilized SUIT2 cells engineered to express AST factors (IKZF1, NFE2, IRF8) under doxycycline control (SUIT2‐3AST^TetR^). Due to the previous failure to form spheroids from metastatic MDA‐MB‐231 single cells (Figure S11), we pre‐engineered the metastatic SUIT2 spheroids (100 cells per spheroid) prior to encapsulation within the granular scaffolds to model cellular migration (Figure 5A). Unlike HEK293A‐4AST^TetR^, SUIT2 cells exhibited robust migration without AST induction, enabling direct comparison of the efficacy of adherent (SUIT‐A) and AST‐induced (SUIT‐S) migration modes (Figure 5B). In traditional “bulk” hydrogels these cells exhibited mechanical confinement and limited migration (Figure 5C). In fragmented GelMA scaffolds, migration was comparable between SUIT‐A and SUIT‐S cells (*p* > 0.05) indicating that AST induction did not significantly alter migratory capacity (Figure 5D–G). Instead, scaffold stiffness had a stronger influence, with cells in stiffer environments (∼13.3 kPa) exhibiting increased motility, consistent with earlier observations using MDA‐MB‐231 (Figure 3I,J). Notably, AST‐expressing SUIT2 cells displayed migration velocities comparable to AST‐induced HEK cells, suggesting convergence toward similar migratory phenotype (indicated by pink line) (Figure 5G). These results indicate that AST may play a context‐dependent role in cellular migration. In this study, AST induction in the aggressive cancer cell, SUIT2, did not enhance migration compared to adherent cells. Different modes of cell migration are known to require distinct signaling pathway and environmental constraints [41, 42, 43]. In particular, amoeboid migration typically requires higher levels of confinement to facilitate retrograde actin flow and efficient migration [44]. When we look at the interstitial spaces created in the granular platform, the images suggested low level confinement throughout the scaffold (Figure 5H). Our platform generated interstitial widths ranging from 2 to 160 µm (Figure S2), with average diameters of ∼39 and 44 µm (soft and stiff, respectively). In a previous report, immune cells activated ameboid‐like migration (lowly adherent migration) in situations where traditional anchorage‐dependent migration was inhibited due to very dense matrices [45]. Previously, 3–5 µm was reported as the width required for this transition, unlike 7–10 µm which utilized mesenchymal migration [46]. This may explain why the low‐ to non‐adhesive AST phenotypes did not exhibit enhanced migration within this granular scaffold. Overall, these findings suggest that AST contributes to cellular plasticity but does not universally enhance migration, highlighting the importance of environmental context in regulating migrational behavior.

### 2.5 AST Alters Mechanosensitivity in Both Non‐Metastatic and Metastatic Cells

**FIGURE 6:**
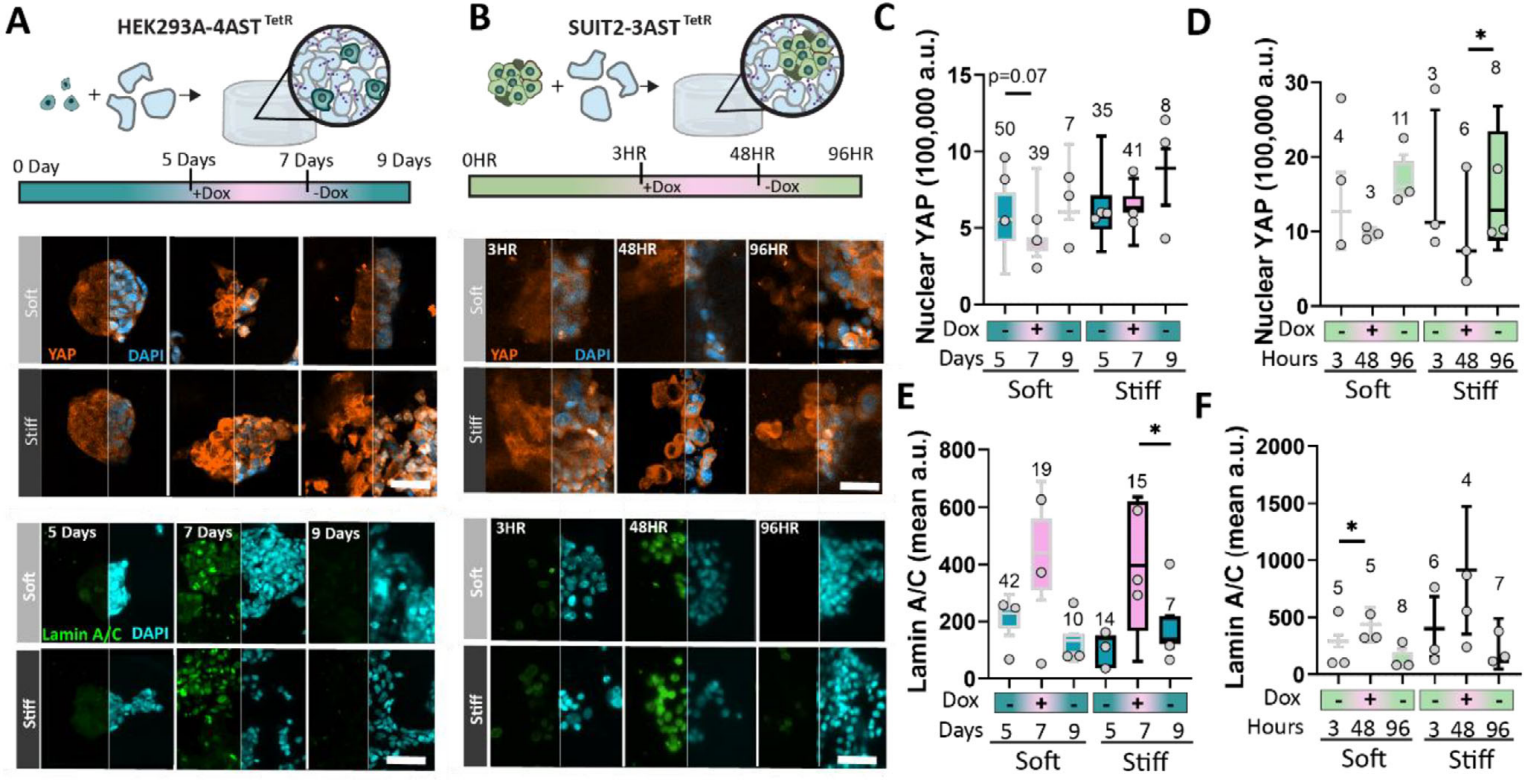
AST induction in non‐metastatic HEK293A‐4AST^TetR^ and metastatic SUIT2‐3AST^TetR^ has an inverse relationship to YAP and elevated Lamin A/C. (A and B) The single‐spheroid growth model was used for HEK293A‐4AST^TetR^ and the pre‐engineered migration model used for SUIT2‐3AST^TetR^. Immunostaining of YAP (orange) and Lamin A/C (green) was performed at time points 5, 7, and 9 days for HEK293A‐4AST^TetR^ (blue) and 3, 48, and 96 h for metastatic SUIT2‐3AST^TetR^ (green). The expression of these proteins in the nucleus was calculated via DAPI segmentation (C–F). (D) Within the stiff scaffold SUIT2 cells in a suspension phenotype had lower nuclear YAP (*p* < 0.05), whilst Lamin A/C expression was enhanced in the soft scaffold (*p* < 0.05) (F). HEK293A also displayed enhanced Lamin A/C expression in the suspension state (*p* < 0.05) (E). Stiffnesses are represented as either a light grey border (soft) or dark grey (stiff). Scale bar represents 50 µm. Box plots represent individual points (number reported above bars), and unless stated a Kruskal–Wallis test or unpaired nonparametric *t*‐test were performed (on non‐parametric data) using the mean of each independent biological repeat (grey dots, *n* = 3). n is equal to the number of spheroids analysed and the field of view (∼20 cells per view) for HEK293A‐4AST^TetR^ and SUIT2‐3AST^TetR^, respectively.

As mentioned earlier, Lamin A/C and YAP are commonly used mechanomarkers and highly implicated in cancer because of its dysregulated ECM. We examined expression of these proteins across the adherent‐to‐suspension transition and the reverse. The HEK293A‐4AST^TetR^ growth model and SUIT2‐3AST^TetR^ migration model were used with doxycycline‐induced AST expression (Figure 6A,B). Across both cell types and scaffold conditions, AST induction was associated with reduced nuclear YAP expression (0.78‐fold, HEK‐S; 0.63‐fold, SUIT‐S) (Figure 6C,D and Figure S12). YAP is a master transcriptional regulator of cancer, enabling phenotypic plasticity [47] via enhanced cell‐ECM interactions and transcriptional programs linked to Hippo pathway [48, 49, 50]. Therefore, it is unsurprising that induced AST expression and sustained anchorage‐independent phenotype (reduced cell‐ECM) is negatively correlated with nuclear YAP expression [14]. Consistent with previous reports, our data supports the necessity of YAP nuclear suppression for transition to a suspension phenotype. Both cell types also showed significantly increased Lamin A/C expression in suspended phenotypes (Figure 6E,F). This aligns with previous RNA sequencing performed on HEK‐S that showed increased LMNA when compared to HEK‐A [14]. Previous findings link Lamin A/C to nuclear stability and survival under mechanical stress, particularly in circulating tumor cells (CTCs) [51]. Elevated Lamin A/C may therefore support cell survival in suspension [51] however, depleted levels are necessary for adhesion and reattachment [52], ultimately supporting the notion that Lamin A/C expression is transient [53]. Together, these results suggest that AST is accompanied by coordinated regulation of mechanosensitive pathways, with reduced YAP activity and increased Lamin A/C expression supporting the transition to a suspension and anchorage‐independent phenotype. This dynamic regulation may facilitate the reversible shifts between adherent and suspended states during metastasis.

## 3 Conclusion

A remodeled TME is strongly associated with early metastatic onset. However, our understanding of this dynamic interplay remains limited by the lack of biomaterials that faithfully recapitulate in vivo conditions. Here, we propose a granular GelMA scaffold with independently tunable stiffness and interstitial spaces that better mimic TME remodeling during early metastasis. This approach overcomes limitations of conventional bulk hydrogels, where mechanical confinement and sub‐microscale pore networks restrict cell behavior. Using this platform, we demonstrated its capability to model tumor growth and cellular migration with two of the most widely used breast cancer cell lines, MCF7 and MDA‐MB‐231. Furthermore, we introduce a novel paradigm, adherent‐to‐suspension transition, which captures non‐metastatic cell migration from a primary spheroid using HEK293A cells, a phenomenon not previously explained by the traditional EMT model. We also reveal mechanosensitive changes in Lamin A/C and YAP associated with both early metastasis and metastatic migration using pancreatic cancer cells, SUIT2. Overall, this study underscores the importance of biomaterial design in cancer modeling and highlights the need to explore AST as a complementary mechanism to EMT in metastatic progression.

## 4 Experimental Section/Methods

### 4.1 Cell Culture

MCF7, MDA‐MB‐231, HEK293A‐4AST^TetR,^ and SUIT2‐3AST^TetR^ cells were cultured using T75 flasks (Conoco) inside an incubator (37°C with 5% CO_2_) until 80% confluency was reached. HEK293A‐4AST^TetR^ and SUIT2‐3AST^TetR^ are cell lines with transfected AST genes which enable the study of the adhesion‐to‐suspension transition. They were procured from Hyun Woo Park at the Department of Biochemistry, College of Life Science and Biotechnology, Yonsei University, Republic of Korea. The media was changed every 2 days and consisted of 1% (v/v) antibiotic‐antimycotic (anti‐anti; ThermoFisher Scientific) and 10% (v/v) foetal bovine serum (FBS; Life Technologies) in either high glucose Dulbecco's Modified Eagle Medium (hg‐DMEM; 11995073, ThermoFisher Scientific) or RPMI 1640 Medium (RPMI; 11875119, ThermoFisher Scientific) for MDA‐MB‐231 and MCF7 or HEK293A‐4AST^TetR^ and SUIT2‐3AST^TetR^, respectively. To passage cells, 2 mL of 0.25% Trypsin‐ethylenediaminetetraacetic acid solution (Sigma) (w/v) was used to facilitate cell retrieval. Once cell detachment and rounding were observed, 5 mL of Hg‐DMEM or RPMI was added and pipetted over the culture surface to ensure maximum cell recovery. The cell suspension was transferred to a 15 mL tube and centrifuged at 800 rcf for 3 min. The supernatant was discarded, and the cell pellet was resuspended in 1 mL of Hg‐DMEM or RPMI. Cells were counted using a hemocytometer and Trypan Blue (Sigma). The desired cell count (200 000 cells per well) was resuspended in cryomedia (10% dimethyl sulfoxide; DMSO, Merck, and 90% FBS) and kept in liquid nitrogen until needed for experiments.

### 4.2 Induction of Adhesion to Suspension Transition

Media was supplemented with 50 µg/mL Doxycycline Hydrochloride, ready‐made solution (Dox; D3072‐1ML; Sigma–Aldrich) and added to the sample after 3 h encapsulation (SUIT2‐3AST^TetR^) or day 5 (HEK293A‐4AST^TetR^) to induce the suspended phenotype. At day 2 (SUIT2‐3AST^TetR^) and day 7 (HEK293A‐4AST^TetR^), the media was changed, samples washed with PBS, and 3 mL of normal respective media was added to each sample to induce the adhesive phenotype.

### 4.3 GelMA Fabrication

Gelatin Methacryloyl (GelMA) was synthesized as described previously [54]. 10 g gelatin (G1890, Sigma–Aldrich) was dissolved in 100 mL of deionized water at 60°C under constant stirring in a round‐bottom flask. Then, 8 mL of methacrylic anhydride (276685, Sigma–Aldrich) was added dropwise to the gelatin solution, stirred for 2 h at 60°C. The gelatin‐methacrylic anhydride solution was then mixed with preheated deionized water at 60°C and maintained for 1 h. The resulting solution was dialyzed in deionized water at 55°C for 7 days using a dialysis membrane (MWCO, approximately 12–14 kDa, Spectrum Laboratories). Subsequently, the solution was filtered through 6 µm‐sized pores qualitative filter paper (TY1‐110, ADVANTEC), lyophilized, and stored at −20°C.

### 4.4 Fragmented GelMA Formation

The lyophilized GelMA was left in a desiccator for 15 min before being dissolved in PBS and 0.1% Irgacure (Ig2959; Sigma) 2‐Hydrocy‐4’‐(2‐hydrocyethoxy)‐2‐methylpropiophenone (diluted in 100% ethanol). The final working solution was kept in a 37°C water bath overnight, then filtered through a 0.2 µm filter syringe and wrapped in aluminium foil until polymerization. To visualize fragments, 1% w/v Fluorescein isothiocyanate‐dextran (FITC‐Dextran; FD2000S, Merck) was added to the precursor GelMA solution prior to polymerization. The fragmentation method was adapted from a previous paper [18]. 3 mL of GelMA precursor solution was added to a 3 mL syringe and then transferred to a preheated transilluminator (15 W, 350 nm, UV transilluminator, UVP) where gels were exposed to 60 s of 3.2 mW/cm^2^. Variations in UV intensity was accounted for using the formula:

$$\mathrm{Total}\;\mathrm{energy}\;\left(\frac{\mathrm{mWs}}{{\mathrm{cm}}^{2}}\right)=3.2\left(\frac{\mathrm{mWs}}{{\mathrm{cm}}^{2}}\right)\;x\;60\;\left(s\right)=192$$

$$\mathrm{Exposure}\;\mathrm{time}\;\left(s\right)=\frac{192}{\mathrm{UV}\;\mathrm{intensity}}$$

The polymerized GelMA was then extruded through an 18G needle into a 5 mL syringe containing 1 mL of PBS. The fragmented GelMA/PBS solution was then sequentially extruded through a 23G, 27G, and lastly a 70 µm pore membrane. The resulting fragment suspension was then centrifuged at 1800 rcf for 5 min and the supernatant discarded. For transferring the fragments, either 200 µL ART Wide Bore filtered tips (2069GPK, ThermoFisher Scientific) or 1000 µL ART Wide Bore filtered tips (2079GPK) were used.

### 4.5 Coverslip and Well Plate Preparation

To prepare samples for immunocytochemistry (ICC), granular gels were adhered to 15 mm diameter coverslips (Menzel‐Gläser), and for live imaging, granular gels were adhered to a glassbottom six‐well plate (20 mm micro‐well, #1.5 coverglass; P06‐20‐1.5‐N, Cellvis). To prepare the glass surface for gel adhesion, it was first treated in an ozone cleaner (UV/Ozone ProCleaner, BioForce Nanosciences) for 2 min. Next, the surface was treated with a methacrylating solution consisting of 20 mL absolute ethanol, 600 µL glacial acetic acid, and 0.1 µL 3‐(Trimethoxysilyl)propyl methacrylate (440159; Sigma–Aldrich) for 5 min. The surface was then washed with absolute ethanol for 1 min, before being air‐dried. For all other surfaces touching the gel, two drops of dichlorodimethylsilane (DCDMS; 440272, Sigma–Aldrich) was added to avoid sticking.

### 4.6 Spheroid Formation

Due to the metastatic nature of MDA‐MB‐231 and SUIT2‐3AST^TetR^ anti‐adherent Aggrewell plates were used to pre‐engineer spheroids. 0.5 mL of Anti‐Adherence (07010; STEMCELL Technologies) was added to the Aggrewell 400 24‐well plate (34415; STEMCELL Technologies) and centrifuged at 1800 rcf for 5 min to ensure no bubbles. This was left to incubate for 30 min at room temperature before being washed with PBS. Cells were thawed and resuspended at a concentration of 200 000 cells per 1 mL in either Hg‐DMEM (MDA‐MB‐231) or RPMI (SUIT2‐3AST^TetR^). The plate was centrifuged at 800 rcf for 5 min and left to incubate overnight at 37°C for next day encapsulation. To retrieve spheroids, the media was carefully aspirated, then 1 mL of PBS was pipetted up and down three times to dislodge the spheroids. 500 µL of the spheroid suspension was added per 300 µL of GelMA fragments.

### 4.7 Embedding Cells Into Fragmented Gel

300 µL of fragmented GelMA was aliquoted per sample and resuspended in 500 µL of PBS (total 800 µL PBS/fragment suspension). As previously mentioned, MDA‐MB‐231 and SUIT2‐3AST^TetR^ were pre‐engineered into spheroids the day prior to encapsulation. 500 µL of each spheroid suspension was added to the PBS/fragment suspension. MCF7 and HEK293A‐4AST^TetR^ were embedded as single cells to grow as spheroids within the scaffold. The cell suspension was directly added to the fragmented GelMA suspension to equate to 200 000 cells per 300 µL of fragments. For live imaging, H2B‐GFP‐tagged transfected cell lines were used.

### 4.8 Embedding Cells in Bulk Hydrogel

For MDA‐MB‐231 spheroids cultured in bulk hydrogel, a previous method was used for traditional bulk hydrogel fabrication [17].

### 4.9 Granular GelMA Scaffold Formation

The fragment/cell suspension was centrifuged at 3000 rpm, and the supernatant was discarded. Using the wide‐bore tips, 200 µL of granular GelMA with embedded cells was transferred to either a methacrylated glass bottom six‐well plate (for live imaging) or a DCDMS glass bottom six‐well plate (for ICC). Either a DCDMS coverslip or a methacrylated 15 mm diameter coverslip was added. The plate was then transferred to a preheated UV transilluminator and exposed for 120 s (adjusted according to UV intensity as described previously). The coverslips were carefully removed, and 3 mL of respective media was added. Routine cell culture was performed. Dox was added to the respective cell type to study the AST transition.

### 4.10 Atomic Force Microscopy (AFM)

To prepare AFM samples, 20 µL of fragmented GelMA was resuspended in 50 µL of PBS and added to a DCDM glass‐bottom six‐well plate before a methacrylated 15 mm diameter coverslip was added. This results in individual fragments adhering to the coverslip. 3 mL of media was added and samples were kept at 5% CO_2_ and 37°C. AFM measurements were conducted at multiple time points during the experiment to monitor degradation. The stiffness of the hydrogels was determined via surface indentation using an MFP‐3D Origin atomic force microscope (Asylum Research). Pyramid‐shaped 200 µL gold‐coated silicon‐nitride tips were used (PRP‐TR‐20, NanoWorld). The samples were washed with PBS to detach any loose fragments, and then submerged in PBS, and wet measurements were taken. Triple indentations were made on separate fragments using a 2 µm/s approach velocity, 10 µm/s retract velocity, and 2 nN trigger force. The portion of the force curve representing contact with the gel was used for Igor Pro posthoc analysis to determine the gel stiffness (Young's Modulus, kPa), using a method previously described [55]. The average of the triplicate was taken. AFM samples were prepared per independent biological repeat to ensure reproducibility of fabrication methodology.

### 4.11 Quantitative Micro‐Elastography (QME)

We used optical coherence elastography (OCE), specifically QME, to map granular elasticity in 3D [56]. The sample was brought into contact with a rigid glass imaging window and rigid plate, which applied approximately 5% preload strain to the sample. Using an actuator, an additional microscale compressive loading was applied. Optical coherence tomography (OCT) was used to acquire volumetric images. A focused beam of nonionizing, near‐infrared light (a fiber‐based spectral‐domain OCT system (Telesto 220, Thorlabs Inc., USA) was used on the sample before and after the microscale compression by synchronizing the actuator loading with the OCT scan acquisition. The ring actuator was driven in a quasi‐static regime by a 10 Hz square wave collinearly with the OCT imaging beam and synchronized with the acquisition of OCT B‐scans. Local axial stress in the sample was calculated from the preload strain within a compliant silicone layer placed on top of the sample. The stress‐strain relationship of the compliant layer was characterized and from this the sample's local elasticity was calculated.

### 4.12 Live Imaging

Samples adhered to a glass‐bottom six‐well plate were imaged using the ZEISS Cell Discoverer 7 (CD7), equipped with an Axiocam 807 mono Camera. Plan Apochromat 5x/0.7 Objective lens was used with Optovar Castor Tubelens 2.0x, with a resolution of 3216 × 2208. Immersion was air, and z‐stacks of 8.19 um (step size = 0.63 um) were acquired. An oblique module was used to capture brightfield images, and LED‐module 470 nm to capture the H2B‐GFP‐tagged nuclei channel. Channels were imaged in a separate sequence to minimize spectral bleed‐through, and settings were kept consistent across independent biological repeats. The incubator was set to 5% CO_2_ and 37°C. Imaging occurred for either 4 days or 9 days of culture, for MDA‐MB‐231 and SUIT2‐3AST^TetR^ or MCF7 and HEK293A‐4AST^TetR^, and media was changed every 2 days.

### 4.13 Migration Analysis

H2B‐GFP‐tagged nuclei were tracked using Trackmate [57] and exported into Excel for further characterization of speed and velocity. The formulas are as follows:

$$\mathrm{Speed}\;\left(S\right)=\frac{\mathrm{total}\;\mathrm{distance}\;\left(\mu m\right)}{\mathrm{time}\;\left(s\right)}$$

$$\mathrm{Velocity}\;\left(V\right)=\frac{\mathrm{displacement}\;\left(\mu m\right)}{\mathrm{time}\;\left(s\right)}$$

### 4.14 Immunocytochemistry (ICC)

Samples prepared on coverslips were used for ICC and confocal imaging. Between each step, samples were washed 3 times with PBS for 2 min. Samples were fixed using 4% w/v Paraformaldehyde (PFA; Santa‐Cruz Biotechnology) added at a 1:1 ratio to media for 15 min. Samples were permeabilized using 0.1% (w/v) Triton‐X 100 (Sigma) diluted in PBS at room temperature for 30 min. Blocking was done by adding 5% (v/v) goat serum (Sigma) in PBS to the sample and incubating at 37°C for 1 h and no washing was done prior to addition of primary antibody. Primary antibodies were added (1:100 dilution in 2% (w/v) bovine serum albumin (BSA; Sigma in PBS) and incubated at 4°C for 72 h. The following antibodies were used; Anti‐Ki67 antibody (Ki67; ab16667; abcam) Yes‐associated protein (YAP; sc101199; Santa Cruz Biotechnology), LaminA/C (sc‐7292; Santa Cruz). The respective secondary antibodies were added (1:200 dilution in BSA), Alexa Fluor 594 goat anti‐mouse (a150116; Abcam) and Alexa Fluor 594 goat anti‐rabbit (ab150080; Abcam) and incubated overnight at 4°C. Phalloidin‐iFluor 647 (ab176759; Abcam) was added at a 1:1000 dilution in BSA and incubated at room temperature for 2 h. 1:400 5 mg/mL DAPI (D9542; Sigma) dilution in PBS was added to the sample and incubated for 20 min at room temperature. Samples were then mounted on a microscope slide with an 88% Glycerol solution containing 1% (w/v) of FITC‐Dextran and stored at 4°C until imaging with Nikon C2 Upright Confocal.

### 4.15 Confocal Imaging

ICC samples were imaged using the Nikon C2 Upright Confocal with a Nikon C2plus camera with numerical aperture 0.45 and 1054 × 1054 resolution. Captures were taken 200 µm from the top of the sample to minimize signal reduction throughout the sample. For Lamin A/C, captures were taken using 20X (with 2X zoom) with a 1 µm step size. For Ki‐67 and YAP capture were taken using 10X (with 2X zoom) with a 2.9 µm step size. 405 was used to capture DAPI, 488 to capture FITC‐Dextran in interstitial space, 594 to capture protein of interest (Lamin A/C, YAP, or Ki‐67) and 647 for F‐actin. Galvano scans were taken to minimize spectral bleed‐through. The same lazer intensities were used for all independent biological repeats.

### 4.16 Interstitial Porosity Characterization

A glycerol/FITC‐Dextran solution was used to visualize the interstitial space. Segmentation and quantification of the interstitial space was conducted using Fiji [58]. First, an Unsharp mask (radius 100, mask 0.6) was applied before conversion to an 8‐bit image. To create a binary mask, a mean threshold was applied, followed by a Gaussian Blur (sigma = 5) and another mean threshold. This generated smooth masks. Next, using BoneJ [59] Area/Volume Fraction function, we calculated BV/TV ratios, which equate to interstitial volume/total volume. Using BoneJ, thickness maps were then generated from the binarized images. Images were converted to isometric prior to calculation. Histograms detailing all measured pore widths were generated from the thickness maps, pooled across samples, and averaged for each independent biological replicate. A cumulative distribution plot was subsequently generated by calculating the percentage contribution of each width bin relative to the total pore count.

### 4.17 Ki‐67 Measurement

To achieve Ki‐67 ratio (+ve/total nuclei), we used Trackmate [60] with Stardist [61] to generate filtered nuclei (Ki‐67 positive nuclei) labels and all nuclei (total nuclei) labels. First, a background subtraction (radius = 20, no sliding) was performed on the DAPI channel. After the Stardist segmentation was completed, the following filters were applied.

To achieve a total nuclei mask: Based on area, a minimum of 5.00 pixels and an upper boundary of 400.00 pixels.

To achieve Ki‐67 positive cells: Based on area, a minimum of 5.00 pixels and an upper boundary of 400.00 pixels. Based on channel intensity (for Ki‐67) with mean intensity above 1000 (A.U). Using the MorphoLibJ [62] Analyse 3D regions function, the number of nuclei present in the masks (Ki‐67 positive nuclei + total nuclei) was counted.

### 4.18 Segmentation of DAPI and F‐Actin to Generate Masks for Protein Intensity Measurements

Nuclei segmentation was performed using a custom Jython macro utilising the trackmate‐stardist plugins. First, background subtraction is performed on the DAPI channel (radius 20, no rolling). The Stardist detector was used for segmentation and the following filters were applied (spot filters; area > 5.00 and area < 400.00), track filters (number of spots > 3.5). These labels were exported as masks. Nuclei volume of less than 100 µm^3^ was excluded from the dataset.

To generate a secondary mask for total cell volume or spheroid volume, the F‐Actin channel was used. We applied Gaussian blur (2, 2, 2) followed by a mean binary threshold, repeated twice in total. This mask was imported into FOCUST to analyze spheroid morphological data as well as protein intensities. This was also used to generate a tertiary mask (cytoplasmic): the primary mask (nuclei) was subtracted from the secondary mask (whole cell/spheroid). Later used to calculate cytoplasmic YAP intensity and generate YAP nuclear‐to‐cytosolic ratio (nuc/cyto) intensity.

For banding analysis, another mask was generated by applying Gaussian blur (20,20,20) followed by a mean binary threshold, which was repeated for a smooth mask. Using FOCUST, the mask can be segregated into 25% quartiles, whereby the intensity within each band was recorded. FOCUST [63] also makes use of Morpholibj and CLIJ2 [64].

### 4.19 Statistical Analysis

All statistical analysis was performed in GraphPad PRISM. A normal Gaussian distribution was used to test for normality and lognormality. Unless stated, data was not normally distributed and a Kruskal–Wallis test or unpaired nonparametric *t*‐test with a Kolmogorov–Smirnov test was used to detect significant differences between the average of each round. For normally distributed data we used a one‐way ANOVA, or an unpaired parametric test with Welch's correction was used. Two‐way ANOVA was used to compare two groups (i.e., soft and stiff). Multiple comparisons Tukey post hoc tests were performed where appropriate. Differences were considered significant when *p < 0.05, **p < 0.01, ***p < 0.0001, and ****p <0.0001. A minimum of three independent biological replicates were analyzed for each condition. Box plots were generated from individual data points (n), with grey circles representing the mean of each biological replicate. Statistical analyses were performed on the means from each independent biological replicate. Results written in text unless otherwise stated represent mean ± SD.

## Author Contributions

**Kai L. Metzner**: methodology, data curation, investigation, formal analysis. **Zhuang Min Lee**: methodology, investigation, formal analysis, data curation, visualization. **Farzan Navaeipour**: methodology, data curation, investigation, formal analysis. **Sebastian E. Amos**: methodology, software, visualization. **Yu Suk Choi**: conceptualization, methodology, data curation, validation, supervision, funding acquisition, visualization, project administration, resources, writing – original draft, writing – review and editing. **Jong Hwa Byun**: methodology, investigation, validation. **Ji Hoon Jeong**: methodology, resources, validation. **Brendan F. Kennedy**: supervision, funding acquisition, writing – review and editing, investigation, resources. **Jiayue Li**: methodology, data curation, investigation, formal analysis. **Yongsung Hwang**: methodology, validation, supervision, resources. **Hyun Woo Park**: conceptualization, validation, supervision, funding acquisition, resources, writing – review and editing. **Hee Seung Lee**: resources, validation. **Juyeon Kim**: methodology, validation, resources. **Khoon S. Lim**: methodology, validation, resources. **Dong Woo Son**: methodology, investigation, validation. **Danielle Vahala**: conceptualization, methodology, data curation, investigation, validation, formal analysis, visualization, writing – original draft, writing – review and editing.

## Funding

This work was supported by the Australian Government Research Training Program Scholarship (DV, SEA, FN, JL, KLM). UWA RIGG (YSC, HWP), ARC DP220100163 (YSC, BFK), CCWA project grant (YSC, BFK). NAWA Research Chair Program (BFK), the National Science Centre, Poland (BFK). National Research Foundation of Korea (RS‐2025‐18362970 to HWP, RS‐2025‐00556619 to HWP, RS‐2026‐25525017 to HWP), Ministry of Health & Welfare of Korea (RS‐2025‐25459146 to HSL, HWP and YSC and RS‐2026‐25613193 to HWP) and Korea Basic Science Institute (National Research Facilities and Equipment Centre) grant funded by the Ministry of Education (RS‐2025‐02402969 to HWP).

## Conflicts of Interest

The authors declare no conflicts of interest.

## Supporting information




**Supporting File 1**: advs77478‐sup‐0001‐VideoS1.avi.

**Supporting File 2**: advs77478‐sup‐0002‐VideoS2.avi.

**Supporting File 3**: advs77478‐sup‐0003‐VideoS3.avi.

**Supporting File 4**: advs77478‐sup‐0004‐VideoS4.avi.

## Data Availability

The data that support the findings of this study are available in the supplementary material of this article.
